# Development of a visual multiplex fluorescent LAMP assay for the detection of foot-and-mouth disease, vesicular stomatitis and bluetongue viruses

**DOI:** 10.1371/journal.pone.0278451

**Published:** 2022-12-08

**Authors:** Qing Fan, Zhixun Xie, You Wei, Yanfang Zhang, Zhiqin Xie, Liji Xie, Jiaoling Huang, Tingting Zeng, Sheng Wang, Sisi Luo, Meng Li

**Affiliations:** Guangxi Key Laboratory of Veterinary Biotechnology, Key Laboratory of China(Guangxi)-ASEAN Cross-Border Animal Disease Prevention and Control, Ministry of Agriculture and Rural Affairs of China, Guangxi Veterinary Research Institute, Nanning, Guangxi, China; Huadong Research Institute for Medicine and Biotechniques, CHINA

## Abstract

Loop-mediated isothermal amplification (LAMP) is a nucleic acid amplification technique that can be used to amplify target genes at a constant temperature, and it has several advantages, including convenience, specificity and sensitivity. However, due to the special interpretation methods of this technology for reaction results, all the previously reported LAMP detection methods have been restricted to identifying a single target, which limits the application of this technology. In this study, we modified conventional LAMP to include a quencher-fluorophore composite probe complementary to the F1c segment of the inner primer FIP; upon strand separation, a gain in the visible fluorescent signal was observed. The probes could be labeled with different fluorophores, showing different colors at the corresponding wavelengths. Therefore, this multiplex LAMP (mLAMP) assay can simultaneously detect 1–3 target sequences in a single LAMP reaction tube, and the results are more accurate and intuitive. In this study, we comprehensively demonstrated a single-reaction mLAMP assay for the robust detection of three cattle viruses without nonspecific amplification of other related pathogenic cattle viruses. The detection limit of this mLAMP assay was as low as 526–2477 copies/reaction for the recombinant plasmids. It is expected that this mLAMP assay can be widely used in clinical diagnosis.

## Introduction

Nucleic acid amplification technologies are among the most valuable tools in the field of molecular biology and can achieve the detection and quantitative analysis of trace nucleic acids. Thus, these technologies are widely used in application-oriented fields, such as clinical medical diagnosis, animal and plant quarantine, food safety and transgenic detection [1, 2]. Polymerase chain reaction (PCR) is the most widely used technique for nucleic acid amplification, but thermocycling is necessary to separate the DNA double strand, the cooling cycle must be heated repeatedly through amplification, and the amplification results must be analyzed by agarose gel electrophoresis or an expensive fluorescence quantitative PCR instrument, which requires 2–3 hours [3, 4]. This characteristic limits the application of PCR, as the process relies upon specialized laboratories [5].

Currently, preventing and controlling infectious diseases requires efficient point-of-care pathogen detection platforms. The diagnostic platform for field detection should be inexpensive, sensitive, specific, simple, easy to operate, stable and fast; loop-mediated amplification (LAMP) and recombinase polymerase amplification (RPA) technologies should meet those requirements [6–12]. LAMP was first reported in 2000 by Notomi et al. [13]. The LAMP method employs a Bst DNA polymerase with strand displacement activity, and in this method, a set of six specific primers (including loop primers) recognize eight regions on the target sequences to complete amplification at constant temperatures between 60 and 67°C. The LAMP products were analyzed by three methods. The first method was visual inspection of the turbidity of the LAMP products, which was formed by the accumulation of magnesium pyrophosphate byproducts [14]. The second method was the addition of a fluorescent dye (i.e., SYBR and calcein dyes) to observe the color change [15, 16]. Finally, the LAMP products were analyzed by agarose gel electrophoresis, which easily causes aerosol pollution in laboratories and is currently not recommended. The results of positive samples presented by these three analysis methods are the same regardless of single or multiple reactions. It is impossible to identify the targets responsible for positive results, and differentiation of multiple products cannot be achieved, which limits the application of this technology [17–23]. In the clinic, there are fewer single-pathogen infections, whereas most cases are multipathogen coinfections.

In 2012, Tanner et al. [24] demonstrated for the first time that LAMP technology combined with DARQ (detection of amplification by release of quenching) probes could be used to identify and detect multiple target DNAs in one LAMP reaction. This study improved the previous DARQ LAMP assay by adding reverse transcriptase to the reaction system, optimizing the reaction conditions, and using composite probes similar to DARQ probes to develop a visualized multiplex fluorescent LAMP (mLAMP) assay for the simultaneous detection of foot-and-mouth disease virus (FMDV), vesicular stomatitis virus (VSV) and bluetongue virus (BTV) and to evaluate the specificity, sensitivity and clinical applications of this assay.

## Materials and methods

### Ethical statement

This study was approved by the Institutional Animal Care and Use Committee (IACUC) of Guangxi Veterinary Research Institution (GVRI). Sample collection was conducted based on protocol #2020A001 issued by the IACUC of GVRI. All samples were collected from live cattle on approved farms by well-trained veterinarians. All methods were performed in accordance with the relevant guidelines and regulations.

### Principle

**Fig 1:**
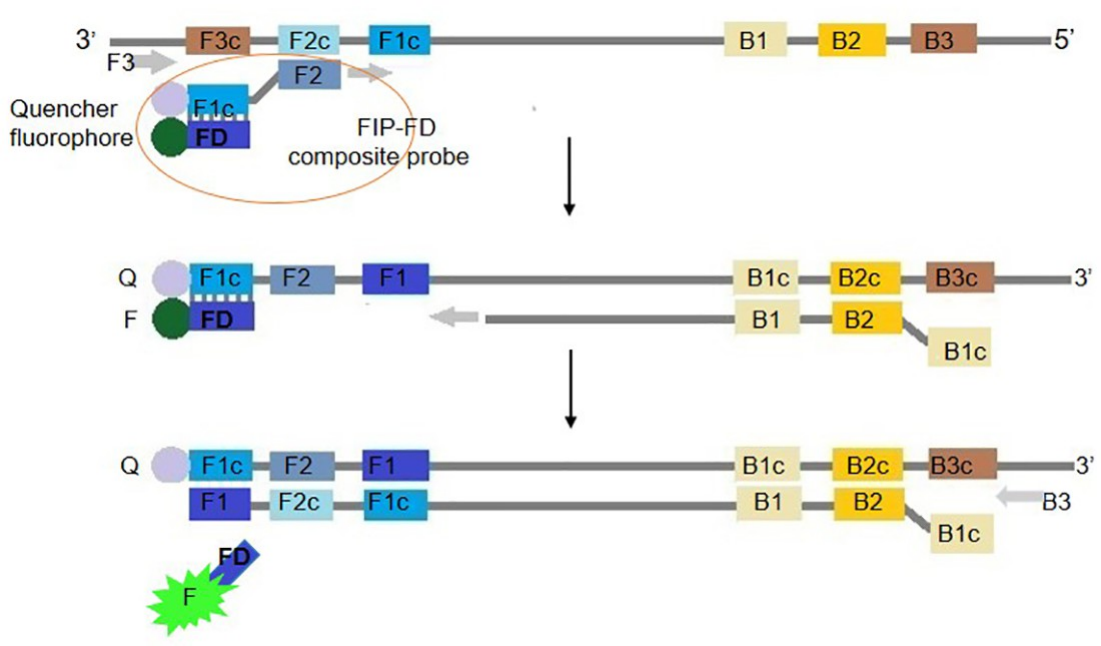
Schematic presentation of the mechanism of mLAMP.

The standard LAMP primers included the outer primers F3 and B3, inner primers FIP (FIP = F1c+F2) and BIP (BIP = B1c+B2), and loop primers Floop and Bloop. Based on the standard LAMP primer, a probe (termed FD) complementary to F1c was synthesized. The FD probe was modified at the 3’ end with a fluorophore, and the FIP was modified at the 5 ’end with a dark quencher. Before the reaction, FIP and FD were annealed to form a quencher-fluorophore composite probe (FD-FIP). Since FD was complementary to the F1c segment of FIP, the fluorophore was close to the quencher, resulting in fluorescence extinction. During the reaction process, FIP retained the function of the inner primer to guide the amplification, but upon synthesis from the reverse direction guided by BIP, the FD probe was separated, causing the fluorophore to release [24, 25]. LAMP is highly efficient and can amplify a few copies of DNA to 10^9^ in less than an hour with greater specificity; thus, a large amount of free FD-fluorophore is released after the reaction, which produces different fluorescent colors under appropriate spectral channels (Fig 1). Multiplex identification tests can be performed according to the fluorescence color with which the FD probe is labeled. Here, we demonstrated a multiplex fluorescent LAMP assay for the simultaneous identification of three different targets in the same reaction. The fluorophores and detection channels are shown in Table 1.

**Table 1:** The fluorophores and detection channels.

Target	FIP-5’	FD-3’	Wavelength (nm)	Color
**1**	FAM/FITC	BHQ1	520	green
**2**	Cy5	BHQ3	670	red
**3**	Cy3	BHQ2	570	blue*

*The color of Cy3 in the spectrum is orange, and the overlapping color of FAM (green) and Cy5 (red) is also orange. To distinguish them, Cy3 is shown in blue in the output image.

### Pathogens and nucleic acid extraction

FMDV serotype A, O and Asia I strains, VSV serotype New Jersey (NJ) and Indiana (IND) strains, BTV serotype 1 and 2 strains, and the viral genomic RNA of peste des petits ruminants virus (PPRV) and epizootic hemorrhagic disease virus (EHDV) were obtained from Yunnan Entry-Exit Inspection and Quarantine Bureau (YNCIQ). The genomic DNA/RNA of bovine viral diarrhea virus (BVDV), swine vesicular disease virus (SVDV), mycoplasma bovis (MB) and infectious bovine rhinotracheitis virus (IBRV) were prepared in our laboratory. Ten nasal swabs and 10 whole blood samples were collected from healthy cattle (FMDV-, VSV- and BTV-free) and were used as negative controls. RNA from the target viruses and negative control were extracted by using the Universal EasyPure RNA Kit (Transgen, China) according to the manufacturer’s instructions and were stored at -80°C until use. Two microliters of DNA/RNA was used as a template for the mLAMP reaction to determine its specificity.

### Primer and probe designs

Multiplex LAMP detection of viral cDNA was investigated by using three primer and probe sets targeting FMDV, VSV and BTV. According to the conserved sequence of the FMDV 3D gene, VSV nucleoprotein N gene and BTV VP7 gene, three sets of specificity LAMP primers and FD probes were designed by using Primer Premier 5 and PrimerExplorer V5 online software (http://primerexplorer.jp/e/) [26–28]. All oligonucleotide primers and FD probes were synthesized by Takara (Takara, Dalian, China), and their sequences are listed in Table 2. The fluorophores FAM, Cy5 and Cy3 were used to label the 3’ end of the FD probe, and the corresponding BHQ series quenching group was used to label the 5’ end of the inner primer FIP. FIP-quencher and FD-fluorophore annealing was performed before the reaction to keep the fluorescence in the quenching state. A mixture of 50 μM FIP and 50 μM FD was heated to 90°C for 5 min and slowly cooled to room temperature to form the FD-FIP composite probe, which was stored at −20°C.

**Table 2:** Primer and FD probe sequences for the mLAMP reaction.

Primer	Target Gene	Sequence (5’-3’)	TM/°C	Final Conc. (μM)
FMDV-F3	3D	GTTTGAGGAGGTGTTCCGC	60.2	0.1
FMDV-B3		CATAGTGTCTACGCAGGGC	59.2	0.1
FMDV-FIP		BHQ1-GTAGGCGTGCTCCGTATTCACAGTTTGGCTTCCACCCGAA	64.2/59.5	0.4
FMDV-BIP		AGGGTGGAATGCCATCTGGTTGGTAGAGCACGTGGATGTTGT	59.5/64.9	0.8
FMDV-Floop		GTCTTCAGAATCCACTCGGCA	61.4	0.2
FMDV-Bloop		TCCGCAACAAGCATTATCAAC	60.3	0.2
FMDV-FD1*		TGTGAATACGGAGCACGCCTAC -FAM	64.2	0.4
FMDV-FD2*		GAATACGGAGCACGCCTAC -FAM	57.1	0.4
FMDV-FD3*		TACGGAGCACGCCTAC -FAM	50.9	0.4
FMDV-FD4*		GGAGCACGCCTAC -FAM	39.4	0.4
VSV-F3	N	GAACTGAAGACAGCACTTC	55.0	0.1
VSV-B3		CCATCCTCGACTAGACTCTC	57.5	0.1
VSV-FIP		BHQ3-GGATGTAGATGGGAAGCCATTTTGATGGGAAATCAGACCCT	60.0/57.2	0.6
VSV-BIP		ACGGATTACAGAAAGAAACTACTGGAAATCTGGTTGACGCCAC	60.5/57.0	0.8
VSV-Floop		ATCCTCCTCAGCAGAACGGTC	60.1	0.2
VSV-Bloop		ACGGGCTTGAAAATCAGTGC	60.1	0.2
VSV-FD		AAATGGCTTCCCATCTACATCC-Cy5	60.0	0.2
BTV-F3	VP7	GATGGTTCATGCGTGCCG	60.8	0.1
BTV-B3		TCACGCCTGCTTGAGTTTG	60.2	0.1
BTV-FIP		BHQ2-CACATCTCCTCTTGCTCCAGCAAGTAACCGCGGTAGTGTGT	64.1	0.4
BTV-BIP		TTCAGGGTCGTAACGACCCCATGAGTTACCCTGCGCCAT	60.2	0.8
BTV-Floop		TCAGTGACACTTGAATCATATCCG	65.1	0.2
BTV-Bloop		TGGAGAAGAATTGAAAACTTCGC	60.4	0.2
BTV-FD		TGCTGGAGCAAGAGGAGATGTG-Cy3	64.1	0.4

* The length of FMDV-FD1 to -FD4 decreases by 3 bases, and the sequence decreases by 1 guanine.

### mLAMP reaction

The 25 μL standard mLAMP reaction mixture contained 2 μL nucleic acid template, 12.5 μL WarmStart Multipurpose LAMP/RT-LAMP 2×Master Mix with UDG (NEB, MA, USA), 1.2 μM FIP, 1.2 μM FD-FIP, 2.4 μM BIP, 0.3 μM F3 and B3, 0.6 μM Floop and Bloop, and 16 U Bst 2.0 WarmStart DNA polymerase, 15 U WarmStart reverse transcriptase. For the multiplex assay, the total primer concentrations corresponded to those described for the standard mLAMP reaction, but each set represented 1/n of the total, in which n is the number of targets; in this study, n was 3. The reactions were performed at 63 °C for 75 min for amplification and 80°C for 5 min for termination by using a real-time turbidimeter (LA-320; Eiken Chemical Co., Ltd., Tokyo, Japan).

The mLAMP products were analyzed by using an image analyzer (Universal Hood III, 731BR01622, Bio-Rad, USA). The reaction results were interpreted according to the fluorescent color of the reaction tube under the corresponding 1–3 channels as follows: green tubes were considered FMDV-positive and were detected in the 520 nm channel (FAM labeled), red tubes detected in the 670 nm channel were considered VSV-positive (Cy5 labeled), and blue tubes detected in the 570 nm channel were considered BTV-positive (Cy3 labeled). Single positive samples exhibited a single color in one corresponding channel, and multiple positive samples exhibited overlapping colors in two or three channels simultaneously. The turbidity curve was generated by a real-time turbidimeter to interpret the process of amplification. The abscissa represents the reaction time, and the ordinate represents the turbidity intensity, which is the amount of white precipitate of the mLAMP byproduct magnesium pyrophosphate. The turbidity curve can detect only positive results but cannot distinguish which target caused positive results.

### Preparation of the RNA standards

The 3D gene of FMDV (GenBank accession no. DQ533483.2), the N gene of VSV (GenBank accession no. M31846.1) and VP7 of BTV (GenBank accession no. AY776331.1) were previously cloned into pEASY-T1, an in vitro transcription vector (Transgen Biotech, China) containing the T7 promoter priming site, generating the recombinant plasmids pEASY-FDMV-3D, pEASY-VSV-N, and pEASY-BTV-VP7. These plasmids were linearized by endonuclease enzymatic digestion using EcoRV. RNA transcription was performed using the In vitro Transcription T7 Kit (Takara, Dalian, China). Then, the RNA was purified using the EasyPure RNA Purification Kit (Transgen Biotech, China). The purified RNA transcripts obtained were then quantified by absorbance at 260 nm using a NanoDrop 2000 (ThermoFisher Scientific, Waltham, USA). Each concentration of the purified RNA transcripts was adjusted to 3.0 ×10^9^ copies/μL and mixed equally to prepare the RNA standards by serial 10-fold dilution with RNase-free H_2_O. The standards contained the three in vitro‐transcribed RNAs with specific gene sequences of the three target viruses, and the concentrations of each RNA ranged from 1×10^8^ to 1 copies/μL. Two microliters of standard RNA transcript was used as a template in the mLAMP assay for analysis of the sensitivity of the mLAMP assay.

### Real-time RT–PCR

Real-time RT–PCR, which is considered the gold standard, was used in parallel for comparison with the performance of mLAMP in clinical detection. The FMDV-specific primers and probe targeting the 3D gene of FMDV, the VSV-specific primers and probes targeting the L gene of VSV, and the BTV-specific primers and probe targeting the NS3 gene of BTV are listed in S1 Table in S1 File. The real-time RT–PCR protocols were carried out by using the Transcript Probe One-Step qRT–PCR SuperMix Kit (Transgen Biotech, China) according to the relevant references [29–31].

### Clinical sample detection

To evaluate the ability of mLAMP to detect viruses in clinical samples, a total of 111 clinical samples, including 12 vesicular fluid samples, 30 esophageal–pharyngeal (OP) samples, 42 whole-blood samples, 6 vesicular skin samples, and 21 oral swabs, were collected from cattle in Guangxi Province, China, between January 2020 and July 2022. RNA was extracted as described above and tested using the mLAMP assay. For comparison, RNAs were also tested using conventional FMDV-, VSV- and BTV-specific single real-time RT–PCR methods [29–31]. All positive clinical samples detected by real-time RT–PCR were confirmed by DNA sequencing to rule out false-positive results. The results of mLAMP and real-time RT–PCR assays were compared by measuring the degree of agreement and kappa coefficient (k).

## Results

### Optimization of the ratio of FD-FIP composite probes to unlabeled FIP primers

**Fig 2:**
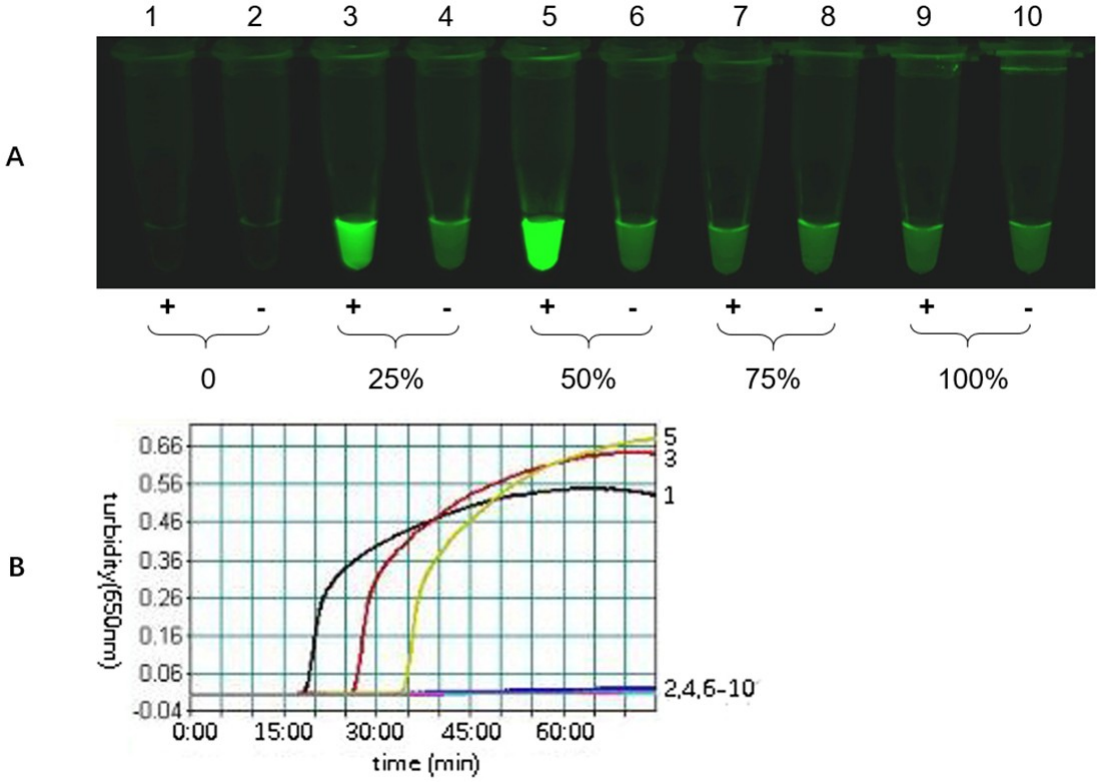
The effect of the ratio of FD-FIP composite probe to unlabeled primer on FMDV amplification. The mLAMP reaction was optimized by using FMDV-specific primers and an FD-FIP composite probe (labeled with FAM and BHQ1). The products were imaged by using the 520 nm channel (A), and the turbidity curve was generated by a real-time turbidimeter (B). The total amount of FIP in each reaction was maintained at 0.8 μM and composed of various ratios of FD-FIP to unlabeled FIP as follows: 1: FD-FIP: unlabeled FIP = 0; 3: 25%; 5: 50%; 7: 75%; 9: 100%, 2, 4, 6, 8, 10 were the negative controls for the corresponding proportions. With the increase in the FD-FIP ratio, the inhibition became more severe with a slower amplification time. At FD-FIP concentrations equal to or higher than 75%, the turbidity curve and fluorescence increase were not observed, suggesting that the reaction was completely inhibited at these concentrations. An equimolar ratio (50%) was used to balance the fluorescence signal and amplification rate for FMDV amplification in the mLAMP reaction.

To enable multiplexing, we utilized the composite probe mLAMP paradigm, which we believe is the most robust and universal approach for multiplexing with LAMP. However, the composite probe is highly inhibitory toward amplification reactions. A previous publication by Tanner reported that when the FD-FIP composite probe completely replaced the FIP primer, a relatively severe inhibitory effect on amplification was observed. This inhibition was significantly reduced using proportionally unlabeled FIP primers and FD-FIP composite probes [24]. The ratio of FD-FIP composite probes to unlabeled FIP primers is the key to the mLAMP assay and is the foremost condition that should be optimized. This study also confirmed this effect and optimized the working ratio of FD-FIP composite probes to unlabeled FIP primers in the mLAMP system for the detection of three cattle viruses. Fig 2 shows the impact of the working ratio of the FD-FIP composite probe to unlabeled FIP primer at 0, 25%, 50%, 75%, 100% for FMDV amplification in the mLAMP reaction. The results show that a working ratio of 50% FAM is the optimal working ratio that could effectively reduce the inhibition effect and produce a clear fluorescence signal to discriminate between negative and positive samples. Similarly, the working ratios of Cy5 and Cy3 were also optimized in the same method as described above, and the results showed that 50% Cy3 and 25% Cy5 were the optimal working ratios in the mLAMP reaction.

### Labeling position of the fluorophore/quencher

**Fig 3:**
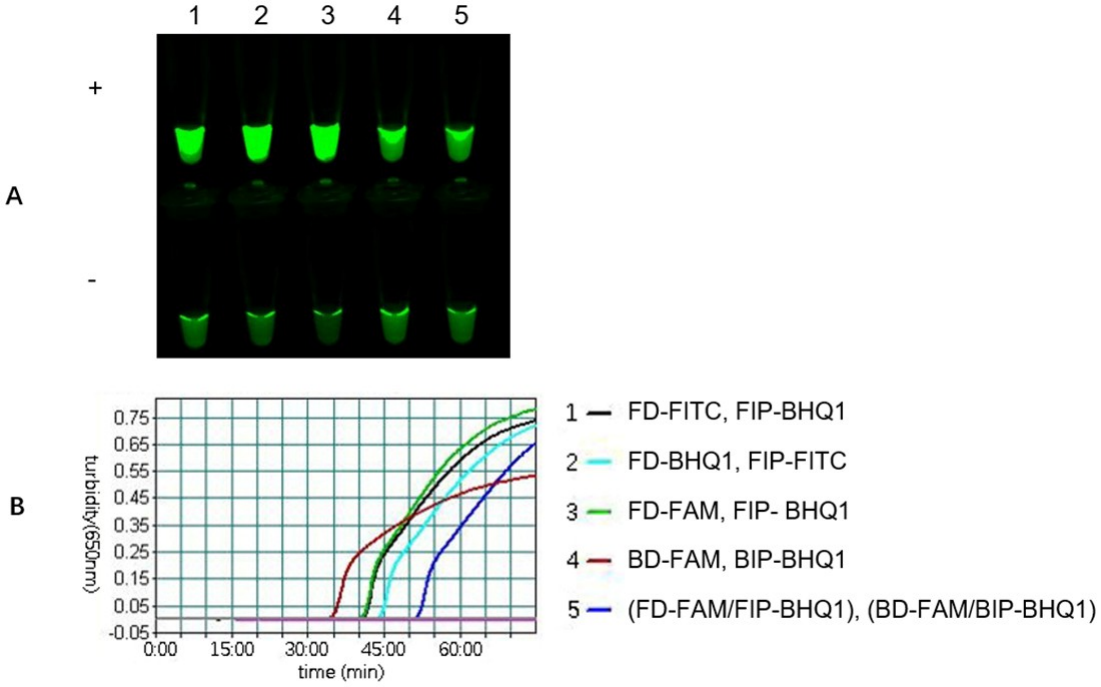
The effect of the labeling position of the fluorophore on the mLAMP reaction. The mLAMP products amplified by using FMDV-specific composite probes with fluorophores/quenchers labeling different positions were imaged using a 520 nm channel (A), and the turbidity curve was monitored by a real-time turbidimeter (B). 1: The FD-FITC/FIP-BHQ1 composite probe is composed of FD labeled with FITC at the 3’ end and FIP labeled with BHQ1 at the 5’ end, and the amplification product showed a robust fluorescence signal with a shorter initial reaction time. 2: For the FD-BHQ1/FIP-FITC composite probe with the quencher and fluorophore position reversed, the initial reaction time was slightly longer than that of composite probe 1. 3: The FD-FAM/FIP-BHQ1 composite probe had a similar fluorescence signal and initial reaction time as composite probe 1. 4: BD-FAM/BIP-BHQ1 composite probe labeled with fluorophore at the BIP terminus. 5: Both the FIP and BIP termini were labeled with fluorophores (FD-FAM/FIP-BHQ1, BD-FAM/BIP-BHQ1). Composite probes 4 and 5 inhibited mLAMP with a weak fluorescence signal compared to that of 1, 2 and 3.

In mLAMP, the composite probe is composed of an FIP primer with a 5’-end labeled quencher and an FD probe with 3’-end labeled fluorescence. To evaluate the effect of the labeling position of the fluorophore/quencher on the mLAMP reaction, we tested the FMDV-specific composite probe with fluorophores/quenchers labeled at different positions (Fig 3). The results showed that the fluorophores/quenchers labeling the FIP terminus (positions 1, 2, and 3) could produce a robust green fluorescent signal, indicating that the fluorophore could be used to labeled either FIP or FD. However, the amplification time of the labeling fluorophore at the FD end (positions 1 and 3) was slightly shorter than that at the FIP end (location 2). The mLAMP reaction was severely inhibited by using fluorophore labeling at the BIP terminal (positions 4 and 5), especially fluorescence labeling at both ends of FIP and BIP (location 5); however, the whole reaction was completely inhibited with very few fluorescence increments that could not be achieved with discrimination between positive and negative samples. In conclusion, the composite probes were successfully synthesized with either a 5′ quencher or the fluorophore FIP if necessary, which allowed for limited modified oligonucleotide synthesis chemistry.

### Shorter lengths of FD provide faster assay times

**Fig 4:**
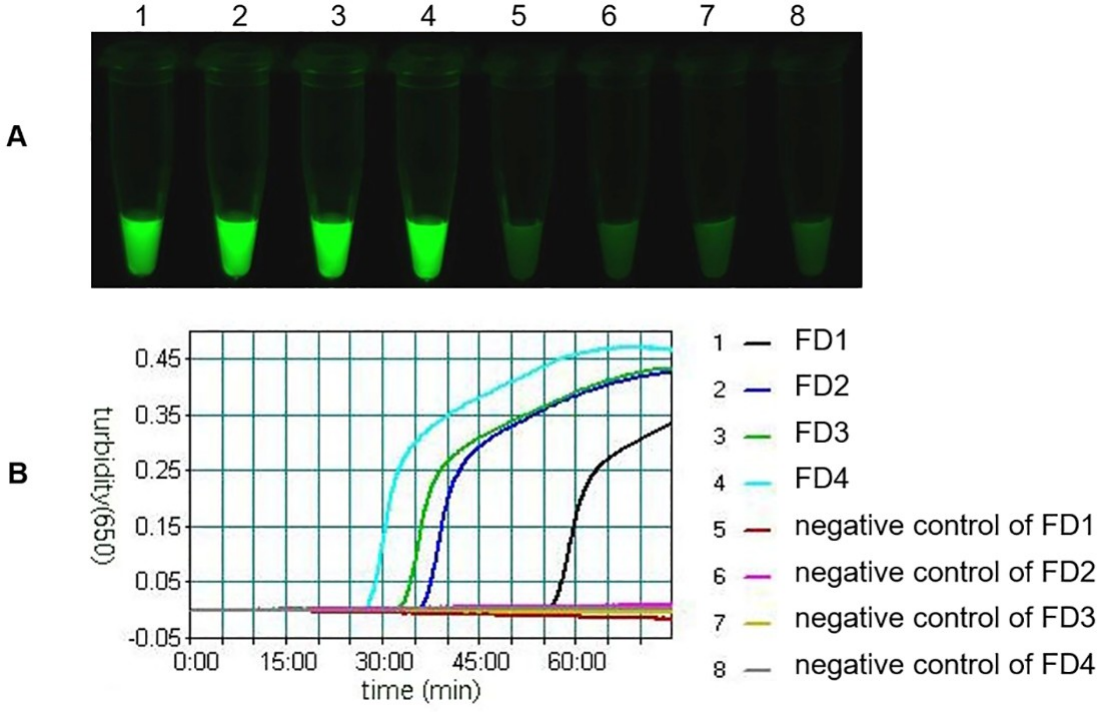
The effect of FD length on the mLAMP reaction. The mLAMP products amplified by using FMDV-specific composite probes with different lengths of FD were imaged using a 520 nm channel (A), and the turbidity curve was monitored by a real-time turbidimeter (B). 1: FD1 consists of 22 bases and is completely complementary to FMDV-F1c with a TM value of 64.2°C, and the initial reaction time is 57.3 min; 2: FD2 consists of removing 3 bases from the 5’ end of FMDV-FD1 with a TM value of 57.1°C, and the initial reaction time is 37.2 min; 3: FD3 consists of removing 6 bases from the 5’ end of FD1 with a TM value of 50.9°C, and the initial reaction time is 34.2 min; 4: FMDV-FD4 consists of removing 9 bases from the 5’ end of FMDV-FD1 with a TM value of 39.4°C, and the initial reaction time is 28.3 min; 5–8: negative controls of FD1-4.

During the LAMP reaction process, the reverse direction synthesis chain guided by BIP separates the FD from the composite probe, releasing the fluorophore. Therefore, the binding force between FD and FIP is the main force leading to the inhibition of the composite probe. The more thymine and guanine (GC) that are present in FD, the more hydrogen bonding between FD and FIP, and the more energy needed for FD to detach from FIP. In the case that GC is greater, the inhibition could be reduced by shortening the FD length. To investigate this, four FMDV-specific FD probes with different lengths were designed to compare the effects of different FD lengths on the mLAMP reaction. The length of FMDV-FD1 to -FD4 successively decreases by 3 bases, and the sequence successively decreases by 1 guanine (or thymine); the parameters are shown in S2 Table in S1 File. The results show that the initial reaction time decreases with the decreasing length of FD1 to FD4, but this decrease in time is unequal. The FD4 probe with the shortest length of 13 bp and a TM value of 39.4°C performed the best and had the shortest initial reaction time (28.3 min). FD1 is completely complementary to 22 bases of FMDV-F1c and has the longest reaction time (the reaction did not start for 57.3 min), and the fluorescence of the FD1 reaction product was slightly weaker than that of the other three probes. Briefly, there were 9 fewer bases in FD4 than in FD1, but reactions with FD4 were 29 min faster than those with FD1 (Fig 4). Overall, shortening the FD probe length is a good strategy for reducing inhibition and shortening the reaction time.

### Bst 2.0 WarmStart DNA polymerase showed more robustness to improve reaction efficiency

**Fig 5:**
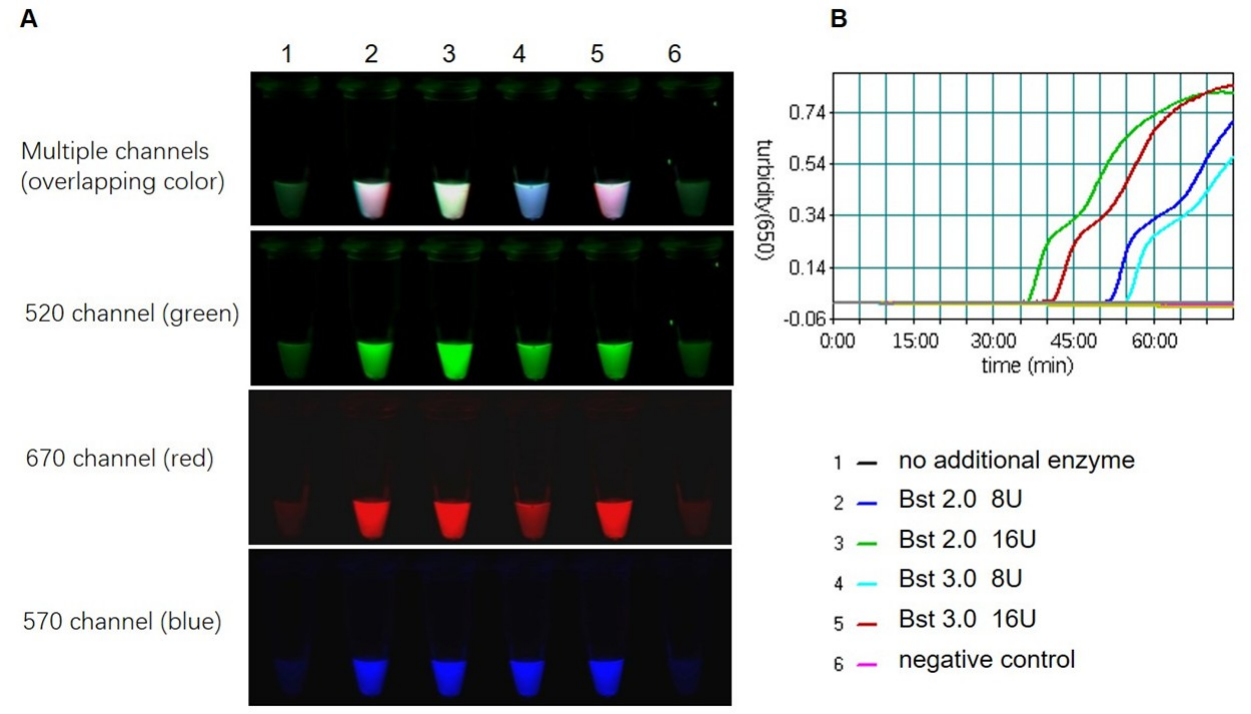
The effect of different strand-displacing enzymes on the mLAMP reaction. The fluorescent mLAMP products were imaged separately using multiple channels (A), and the turbidity was monitored by a real-time turbidimeter (B). Amplification time dependence upon different strand-displacing enzymes with different working amounts. Bst 2.0 WarmStart showed shorter amplification times than Bst 3.0 in the mLAMP reaction. 1: Without addition of enzyme, the reaction was completely inhibited without any amplification; 2: each reaction with 8 U Bst 2.0 WarmStart had an initial amplification time of 52 minutes, 3: each reaction with 16U Bst 2.0 WarmStart had an initial amplification time of approximately 36 minutes, 4: each reaction with 3U Bst 3.0 had an initial amplification time of 55 minutes, 5: each reaction with 16U Bst 3.0 had an initial amplification time of 42 minutes, 6: negative control.

NEB commercial premix contains 8 mM magnesium (Mg^2+^), Bst 2.0 WarmStart DNA polymerase (Bst 2.0 WarmStart) and WarmStart RT× reverse transcriptase. For a single reaction, the premix performed well. However, for multitarget amplification, there were three sets of primers and three composite probes in one reaction system, leading to increased inhibition. The previous section suggested using a 25%-50% ratio of composite probes to unlabeled FIP primers, as the unlabeled FIP primer prevents the reaction from being completely inhibited. However, as one competing reaction component contains three composite probes, these amounts completely inhibited the reaction. The premix performed poorly for multitarget amplification, and an additional enzyme needed to be added to promote multitarget amplification. To determine the optimal enzyme and working amounts, mLAMP reactions containing either Bst 2.0 WarmStart or Bst 3.0 DNA polymerase (Bst 3.0) was used to compare the anti-inhibition effects in mLAMP reactions. The results showed that without the additional enzymes, the mLAMP reaction was completely inhibited with no amplification. With the increase in the working amount of Bst DNA polymerase added, the amplification efficiency increased and the inhibition decreased, which was reflected in the shortening of the initial reaction time and the corresponding increase in fluorescence [25]. In the multiplex assay, better performance was observed for Bst 2.0 WarmStart than Bst 3.0 in NEB commercial premix, in which 16 U of Bst 2.0 WarmStart was added for each reaction with a faster reaction time and higher fluorescence increment (Fig 5). Overall, it is necessary to supplement enzymes in mLAMP for multiple reactions.

### Analytical specificity of mLAMP

**Fig 6:**
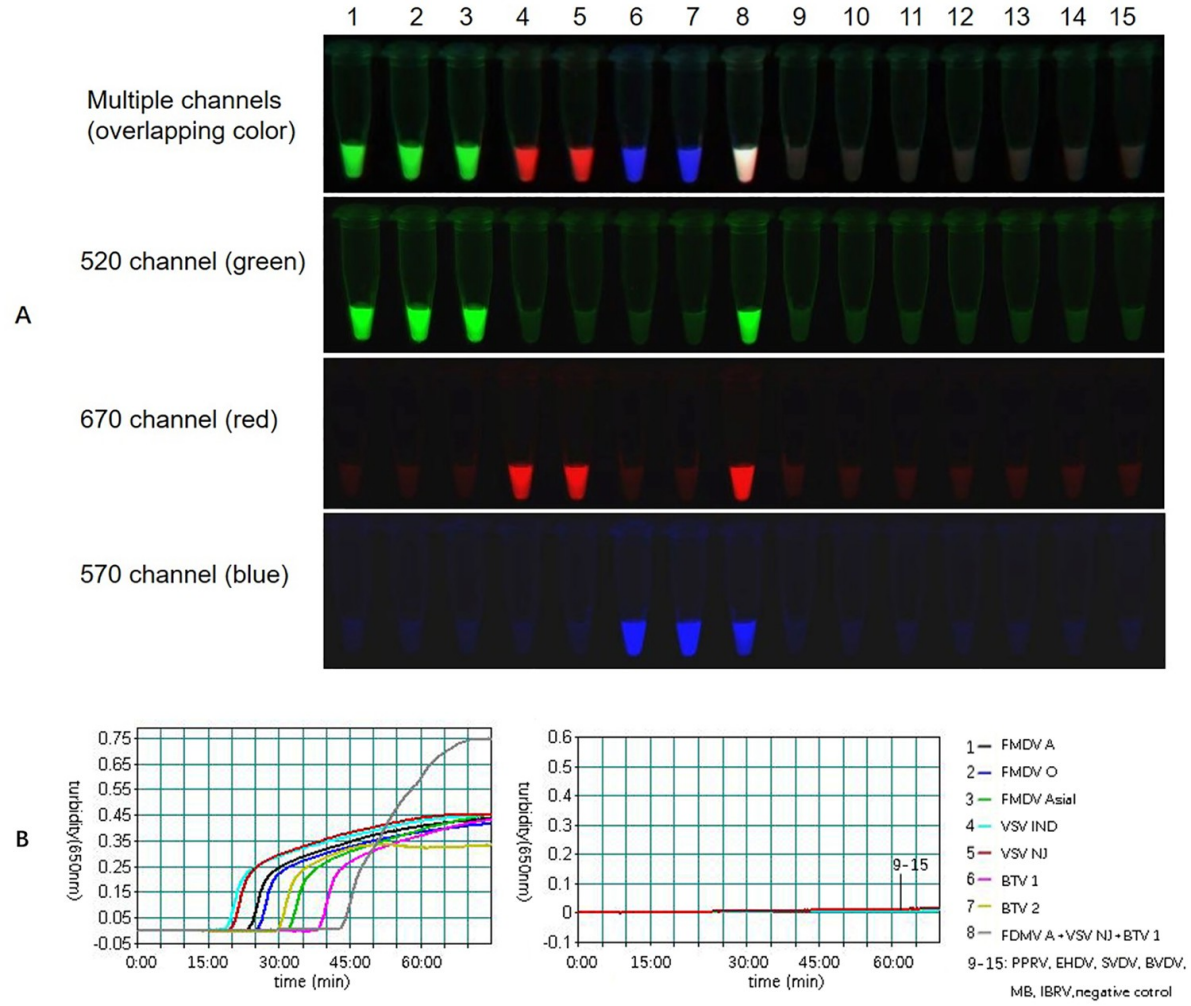
Specificity of mLAMP. The fluorescent mLAMP products were imaged separately using multiple channels (A). The turbidity curve was generated by a real-time turbidimeter to interpret the process of amplification (B). Green fluorescence (FAM) indicates FMDV-positive amplification, red fluorescence (Cy5) indicates VSV-positive amplification, and blue fluorescence (Cy3) indicates BTV-positive amplification. Overlapping fluorescence indicates multiple positive amplifications. Lane 1: FMDV A, lane 2: FMDV O, lane 3: FMDV Asia I, lane 4: VSV IND, lane 5: VSV ND, lane 6: BTV 1, lane 7: BTV 2, lanes 8: FMDV A+ VSV IND + BTV1; 9–15: PPRV, EHDV, SVDV, BVDV, MB, IBRV, negative control.

For a successful multiplex assay, accurately distinguishing the target sequences is crucial. To validate the specificity of the mLAMP assay, we next sought to extend the method to detecting triple targets in a single reaction and to determine whether nonspecific amplification of nontarget template nucleic acids occurred. The mLAMP assay was performed using a single template including 3 serotypes of FMDV (A, O, and Asia I), 2 serotypes of VSV (IND and NJ), 2 serotypes of BTV (1 and 2), 6 other related cattle pathogens (PPRV, EHDV, SVDV, BVDV, MB, IBRV) and a mixed sample containing three target viruses to evaluate its cross-amplification. The results showed that the target viruses and the mixed sample generated a turbidity curve of amplification, but this was not observed for the other related cattle pathogens (Fig 6). Interestingly, the mixed sample containing the three target viruses reacted faster and had a higher turbidity value than that of the single template reaction. This effect was likely due to the turbidity signal in the triple reaction being superposed by multiplex amplification. Each target virus was amplified and showed the corresponding color as expected: the FMDV-positive samples labeled with FAM showed a green color in the 520 channel, the VSV-positive samples labeled with Cy5 showed a red color in the 670 channel, and the BTV-positive samples labeled with Cy3 showed a blue color in the 570 channel. The fluorescence of a single template is visible only in its particular spectral channel and not in other channels. The mixed samples of the three viruses exhibited an overlapping color, which can be observed under the three channels simultaneously. However, tests for the other reference cattle pathogens that are commonly found in cattle were negative, and no cross-amplification was observed [32]. Both the turbidity and fluorescence results indicated that mLAMP has good specificity for detecting FMDV, VSV and BTV without any nonspecific amplification.

### Analytical sensitivity of mLAMP

To determine the detection limits of mLAMP, each 10-fold serial dilution of the RNA standards was subjected to mLAMP with 10 replicates, and the results are shown in S3 Table in S1 File. The detection limit of mLAMP for the three varieties was determined by using probit regression analysis (SPSS, Inc., Chicago) [33]. The SPSS statistical program generated the probit (predicted proportion positive) versus the template concentration with 95% confidence intervals (CI), as shown in Table 3. The detection limit of the mLAMP assay was 2477 copies/reaction for FMDV, 526 copies/reaction for VSV and 913 copies/reaction for BTV. The previous publication showed that the detection limits of conventional real-time RT–PCR were as follows: 11 copies/reaction of the cloned plasmid containing the 3D gene of FMDV, 10 copies/reaction of the cloned plasmid containing the L gene of VSV, and 200 copies/reaction of the cloned plasmid containing the NS3 gene of BTV [29–31]. The sensitivity of this mLAMP for the three cattle viruses was slightly lower than that of conventional single real-time RT–PCR. The reason for this might be that the three sets of primers and probes, with a total of 18 primers and 3 probes, used in the mLAMP assay could compete for the reagents in one reaction and interfere with other amplifications, resulting in reduced amplification efficiency.

**Table 3:** Probit analysis to determine the detection limit of mLAMP.

Probit^a^	Detection limit, copies/reaction (95% CI)		
	FMDV	VSV	BTV
**0.95**	2477(1121–29144)	526(227–14160)	913(368–49718)

^a^ Probit, the predicted proportion of replication from SPSS software, was calculated based on 10 replicates of 9 standards listed in S3 Table in S1 File.

### Comparison of mLAMP and real-time RT–PCR assays in clinical detection

To assess the applicability of mLAMP for the detection of viral RNA in the field, we compared this method with real-time RT–PCR for 111 clinical specimens, and the detection results are shown in Table 4. The positive real-time RT–PCR products were sent to the BGI (Beijing Genomics Institute, China) for DNA sequence confirmation with real-time RT–PCR primers. The sequencing results showed that all the positive results were true positives. The sensitivity and specificity of mLAMP for the detection of BTV were 100% (15/15) and 100% (96/96), respectively, compared to those of the real-time RT–PCR assay. However, in the detection of FMDV, 21 samples were positive by mLAMP, and 22 samples were positive by real-time RT–PCR. One discrepant sample was found, which was positive for FMDV by real-time RT–PCR but negative by mLAMP. The sequencing results also indicated that the discrepant sample was a true FMDV-positive sample. The sensitivity and specificity of mLAMP for the detection of FMDV were thus 95.5% (21/22) and 100% (89/89), respectively. Since VSV has not been found in China thus far, no VSV-positive samples were detected in this test. No coinfected positive samples were detected in this test. Although no VSV-positive samples were detected due to the geographical limitations of sampling, the results for VSV were consistent with those obtained by real-time RT–PCR. The mLAMP results exhibited 99.1% agreement (kappa coefficient, k = 0.98) with the real-time RT-PCR data (S4 Table in S1 File). The results showed that mLAMP had a positivity rate that was similar to that of real-time RT-PCR.

**Table 4:** Performance of the mLAMP assay for detection in clinical samples.

Clinical sample	Number	MLAMP/real-time RT–PCR/sequencing		
		FMDV	VSV	BTV
Vesicular fluid	12	6/6/6	0	0
OP samples	30	9/10/10	0	0
Whole blood samples	42	0	0	15/15/15
Vesicular skin samples	6	6/6/6	0	0
Oral swabs	21	0	0	0
Total	111	21/22/22	0	15/15/15

## Discussion

Based on the previously developed DARQ LAMP assay for the detection of multiple target DNAs, in this study, we successfully developed a visual mLAMP assay for the identification and detection of three bovine viral RNAs by using composite probes. The analytical sensitivity and specificity and the clinical sample detection ability showed that this technique with composite probes was feasible and could be used for the differential detection of multiple RNA targets. Compared with the DARQ assay, which relies on a real-time instrument to read the reaction results, our mLAMP assay simplifies the method for interpreting the reaction results via observation of the color of the reaction tube after the reaction. This method is more intuitive and convenient than the DARQ assay. However, the composite probe is highly inhibitory to amplification reactions; thus, additional studies are necessary to broadly apply the composite probe. Here, we also demonstrated four strategies to reduce the inhibitory effect of the composite probes, providing new ideas and solutions for the establishment of mLAMP diagnostic methods in the future.

First, using a ratio of FD-FIP composite probes to unlabeled FIP primers significantly reduced the inhibition effect. Although the fluorescence of the FD-FIP composite probe was extinguished before the reaction, the composite probe still had a certain fluorescence background value. The FD amount was positively correlated with the fluorescence background value, and a higher FD amount was associated with a higher fluorescence background value. As the amount of FD increased, we observed a gradual increase in inhibition and amplification time. Moreover, a high fluorescence background value is unfavorable for discriminating between negative and positive samples after the reaction has occurred, resulting in inaccurate interpretation of the results. In the multiplex assay, each ratio of FD-FIP composite probe to unlabeled FIP primers is the foremost condition and should be first optimized to screen the optimal working ratio. In this study, a 25%-50% FD ratio was optimized to effectively reduce the inhibition effect and generate a clear fluorescence signal after the reaction to discriminate between negative and positive samples.

This effect was likely due to faster target generation with FIP and easier incorporation with the composite probe during exponential amplification, and using a certain amount of composite probe maintains rapid threshold detection with a high fluorescence signal amplitude. This strategy is important for detecting amplification in mLAMP.

Second, increasing the enzyme amount in multiple reactions improves the reaction efficiency and alleviates inhibition. NEB’s Bst 2.0 WarmStart and Bst 3.0 were developed not only to provide faster amplification times but also to provide robustness against inhibitors. The processivity and anti-inhibition effects of these two enzymes were related to the Mg^2+^ content, reaction buffer components and ratio of the FD-FIP composite probe. Previous publications on mLAMP suggested that using Bst 3.0 resulted in a greater robustness against probe inhibition than that of Bst 2.0 WarmStart at a Mg^2+^ concentration of 3 mM [25]. However, in this study, Bst 2.0 WarmStart exhibited a better anti-inhibitory effect than Bst 3.0 in NEB’s commercial premix with 8 mM Mg^2+^. The results of this study are not consistent with previous publications, but this does not indicate that Bst 2.0 WarmStart is better than Bst 3.0. The optimal enzyme for mLAMP needs to be determined by experimental tests. The primers and probes used in this study combined with the concentration of each component in NEB’s commercial premix (Mg^2+^ 8 mM) were more suitable for Bst 2.0 WarmStart. Most likely, the differences between Bst 2.0 WarmStart and Bst 3.0 resulted from the design changes created during the directed evolution of the enzyme and the relationship of varying buffer components (such as monovalent cations and the amount of Mg^2+^), the melt temperatures for the primers and composite probes, and the resulting processivity of the enzyme. How this impacts the thermodynamic properties of the primer sets in combination with these engineered polymerases is uncertain, i.e., the influence of higher and lower Mg^2+^ amounts on the multiplex assay. The relationship of these properties could be further examined in future studies to better inform optimal primer sets and probe conditions.

Third, compared to the probe designed to label the BIP terminus, the composite probe designed to label the FIP terminus promoted the reaction better. Utilizing composite probes that were synthesized with either a 5′ quencher or fluorophore in the FIP orientation resulted in no difference in amplification detection efficiency or inhibition levels. However, labeling the composite probe in both the FIP and BIP orientations completely inhibited the whole reaction. This is due to the superposition of FIP and BIP bidirectional probe inhibition; as a result, FD was not detached from the composite probe. Although the turbidity curve could be monitored, the fluorescence increment was too small to distinguish the positive and negative samples. The mLAMP reactions perform robust amplification of the three targets, with increment of fluorescence signal accompanying decreased concentration of the fluorophore composite probes. This visible increment of the fluorescence signal emphasizes the need for bright fluorophores with high quantum yield and appropriate spectral matching with the fluorescence detection channels. The LAMP-assisted composite probe accommodates any fluorophore that can be quenched and detected, but brighter fluorophores are preferred when detecting more than three targets simultaneously. In these mLAMP reactions, FAM/FITC, Cy5 and Cy3 are bright fluorophores and provide a sharp contrast of fluorescence signals, which are easy to distinguish from background fluorescence.

Finally, an additional strategy for reduced inhibition is shortening the FD length. The main reason for the inhibition of the composite probe was the hydrogen bonding between FD and FIP, which hindered the separation of FD from FIP by the BIP-guided synthesis chain. Therefore, shortening the FD length could reduce the number of GCs in FD, thus reducing the inhibition effect and promoting the reaction. While the modified FD probe is universal for any LAMP, it is important to note that the design of the FD probe is not straightforward. FD should not be too short; otherwise, it cannot form a stable composite probe with FIP, and fluorescence shedding before the reaction will lead to inaccurate detection results. The TM value is related to the length of FD, and a short FD has a lower TM value. The LAMP reaction temperature is usually 60–67°C, and the design of an FD probe should consider that FD and FIP completely anneal at this temperature without separation. Therefore, each FD probe with different lengths must be validated to screen out the optimal FD, and often, several FD probes must be designed given the complexity.

The composite probe detection assay provides a universal method for multiplex target detection of LAMP amplification without the need for additional primer or probe design; only the addition of a complementary oligonucleotide FD probe to the conventional LAMP primer is needed. After being synthesized from the reverse direction with guidance by BIP, the FD-FIP composite probe is detached, and the fluorophore is released. This detection methodology may be extendable to other established single-target LAMP methods by the addition of an FD “tail” to an FIP primer in the reaction. The established single-target LAMP method can be modified and combined into a multiplex target LAMP method by using a composite probe. Each mLAMP assay must be optimized for each designed primer set, and each ratio of FD-FIP composite probe, enzyme amounts, and amplification time can vary widely in different assays.

## Conclusions

This study combined LAMP technology with a composite probe to develop a visual multiplex fluorescence LAMP assay for the simultaneous identification of multiple target sequences to address the need for point-of-care detection of multiple pathogens.

## Supporting information

S1 File (DOCX)
