# Supplementary material for: Development of a visual multiplex fluorescent LAMP assay for the detection of foot-and-mouth disease, vesicular stomatitis and bluetongue viruses
Source: PLoS One. 2022 Dec 8;17(12):e0278451. doi: 10.1371/journal.pone.0278451 (PMC9731490; doi:10.1371/journal.pone.0278451)
Supplement: S1 File — (DOCX) [file pone.0278451.s001.docx]

**S1 Table. Primers and probes used in real-time RT‒PCR assays.**

| **Primer set** | **Target** | **Sequence (5’-3’)** | **References** |
| --- | --- | --- | --- |
| FMDV-F | 3D | ACTGGGTTTTACAAACCTGTGA | Wang, et al., 2020,  OIE-recommended |
| FMDV-R |  | GCGAGTCCTGCCACGGA |  |
| FMDV-probe |  | FAM-TCCTTTGCACGCCGTGGGAC-BHQ1 |  |
| VSV-F | L | CAGTACAATTAYTTTGGRACMTTTG | Zang, et al., 2015 |
| VSV-R |  | GAGACTTTCTGTYACGGGATCT |  |
| VSV-IND-probe |  | FAM-GACTCTTGTTGATGATGCA-MGB |  |
| VSV-NJ-probe |  | FAM-TGCATGACCCCGCAATCCGGC-MGB |  |
| BTV-F | NS3 | AARGCGGAGAARGCTGCAT | Mulholland, et al., 2017 |
| BTV-R |  | ARYCTGACRTCATCACGAAACG |  |
| BTV-probe |  | FAM-CGCATCGTACGCRGAA-MGB |  |

**S2 Table.** [**Parameter**](javascript:;) [**information**](javascript:;) **for FMDV-FD1 to FMDV-FD4.**

| **Primer** | **Length (bp)** | **TM (℃)** | **GC%** | **ΔG (kcal/mol)** | **Activity (µg/OD)** |
| --- | --- | --- | --- | --- | --- |
| FMDV-FD1 | 22 | 64.2 | 54.5 | -42.6 | 32.0 |
| FMDV-FD2 | 19 | 57.1 | 57.9 | -37.3 | 31.5 |
| FMDV-FD3 | 16 | 50.9 | 62.5 | -32.3 | 32.6 |
| FMDV-FD4 | 13 | 39.4 | 69.2 | -26.4 | 32.8 |

**S3 Table. Number of positive samples, with 10 replicates for each standard dilution.**

| **FMDV Standards^a^ (copies/μL)** | **No. of FMDV positive samples** | **VSV Standards (copies/μL)** | **No. of VSV positive samples** | **BTV-4 Standards (copies/μL)** | **No. of BTV-4 positive samples** | **No. of replicates** |
| --- | --- | --- | --- | --- | --- | --- |
| 1×10^9^ | 10 | 1×10^9^ | 10 | 1×10^9^ | 10 | 10 |
| 1×10^8^ | 10 | 1×10^8^ | 10 | 1×10^8^ | 10 | 10 |
| 1×10^7^ | 10 | 1×10^7^ | 10 | 1×10^7^ | 10 | 10 |
| 1×10^6^ | 10 | 1×10^6^ | 10 | 1×10^6^ | 10 | 10 |
| 1×10^5^ | 10 | 1×10^5^ | 10 | 1×10^5^ | 10 | 10 |
| 1×10^4^ | 10 | 1×10^4^ | 10 | 1×10^4^ | 10 | 10 |
| 1×10^3^ | 10 | 1×10^3^ | 10 | 1×10^3^ | 10 | 10 |
| 100 | 0 | 100 | 6 | 100 | 8 | 10 |
| 10 | 0 | 10 | 0 | 10 | 0 | 10 |
| 1 | 0 | 1 | 0 | 1 | 0 | 10 |

^a^ The standards were in vitro-transcribed RNA with the specific gene sequences of FMDV, VSV and BTV. Two microliters of standard was used as a template in the mLAMP assay.

**S4 Table. Comparison of real-time RT-PCR and mLAMP for viral detection in clinical samples.**

|  | Real-time RT-PCR + | Real-time RT-PCR - | Total |
| --- | --- | --- | --- |
| mLAMP + | 36 | 0 | 36 |
| mLAMP - | 1 | 74 | 75 |
| Total | 37 | 74 | 111 |
|  | Arg: 99.1% | K: 0.98 |  |

+: positive; -: negative; Arg: agreement; K: kappa coefficient.

Agreement=[real-time RT-PCR positive, mLAMP positive + real-time RT-PCR negative, mLAMP negative]/total number of samples.

kappa coefficient was calculated by SPSS software.
